# The role of natural products and traditional Chinese medicine formulas in postpartum depression: mechanism and prospects

**DOI:** 10.3389/fphar.2025.1686499

**Published:** 2025-12-02

**Authors:** Ruirui Shang, Yitong Lu, Haonan Gao, Xia Zhong, Xiaowen Yu, Guoqiang Li

**Affiliations:** 1 College of Rehabilitation Medicine, Shandong University of Traditional Chinese Medicine, Jinan, Shandong, China; 2 First Clinical Medical College, Shandong University of Traditional Chinese Medicine, Jinan, China; 3 Institute of Child and Adolescent Health, School of Public Health, Peking University, Beijing, China; 4 Division 4 of Neurology Department, Affiliated Hospital of Shandong University of Traditional Chinese Medicine, Jinan, China; 5 Department of Pain Medicine, The First Affiliated Hospital of Shandong First Medical University and Shandong Provincial Qianfoshan Hospital, Jinan, China

**Keywords:** postpartum depression, traditional Chinese medicine, natural products, molecular mechanisms, action targets

## Abstract

Postpartum depression (PPD) is a common postpartum complication mediated by multiple factors, which can lead to dual damage to both maternal and infant health. There is an urgent need to explore alternative intervention strategies, as the current conventional antidepressant medications have drawbacks, including delayed onset, severe side effects, and low patient tolerance. Due to their multi-target potential, certain metabolites derived from traditional Chinese medicine (TCM) and other natural products are being investigated as treatments for PPD. However, a systematic understanding of their molecular mechanisms, grounded in the pathophysiology of PPD, is lacking. Therefore, this article synthesizes recent literature to systematically review the pathophysiological mechanisms of PPD and to elucidate the molecular mechanisms underlying the therapeutic effects of TCM formulas and natural products. This review also critically discusses the limitations of current research—particularly issues related to standardization and safety—and proposes key priorities for future preclinical studies and clinical translation.

## 1 Introduction

One of the most common postpartum complications is postpartum depression (PPD), which typically manifests within 1 month after childbirth. It is characterized by chronic depression, anger, anxiety, negative emotions, loss of appetite, and, in severe cases, suicidal thoughts and actions (Ushiroyama et al., 2005). In some low- and middle-income countries or regions, suicide within 1 year after childbirth accounts for about 20% of maternal deaths (Lindahl et al., 2005). Furthermore, PPD poses a long-term risk to the behavioral, emotional, and cognitive development of newborns, in addition to negatively affecting the physical and mental health of mothers (Murray, 1992; Murray and Cooper, 1997a; Murray and Cooper, 1997b; Halligan et al., 2007; Feldman et al., 2009). The incidence of PPD is on the rise due to the accelerated pace of modern life and increasing family pressures. Research indicates that PPD is more prevalent in developing countries (15.0%) compared to developed countries (12.0%), with prevalence rates ranging from 5.0% to 26.32% (Liu et al., 2022). Unlike other physiological stages, postpartum women undergo significant changes in hormones, neurotransmitters, and metabolic levels, making the etiology of PPD more complex and distinct from simple depression (Fox et al., 2018).

Conventional antidepressant medications and neurosteroid-based agents (e.g., brexanolone, zuranolone) are current mainstay pharmacotherapies for PPD. However, these medications carry the risk of being transferred to nursing infants (Patterson et al., 2024). In addition, many PPD patients are either not diagnosed in a timely manner, unable to access psychological treatment, or unwilling to take antidepressant medications during pregnancy or lactation due to concerns about the potential impact on their baby. Meanwhile, the therapeutic effects of antidepressant medications typically take several weeks to manifest, and surveys indicate that 15%–30% of patients discontinue their medication prematurely (Gartlehner et al., 2005). Therefore, developing more effective and safer antidepressant pharmacotherapies is an urgent priority. TCM formulas, which are typically complex mixtures of several botanical drugs, are hypothesized to exert their effects via the synergistic actions of their diverse metabolites on multiple pathways (Zhao et al., 2023). Some of these formulas have already been investigated for PPD management. This is supported by the growing body of research on botanical drugs for PPD (Assadi et al., 2011; Dereli et al., 2019) which provides a foundation for further exploring the potential of TCM formulas and natural products in treating this disorder.

PPD has a complex pathophysiology involving the interplay of both social and biological factors. Social causes include excessive stress, marital discord, and single-parent households, while physiological factors encompass immune system abnormalities, neurotransmitter deficits, rapid estrogen decline, and neuroendocrine imbalances (Anokye et al., 2018; Stewart and Vigod, 2019). In recent years, the occurrence and progression of PPD have been linked to pathogenic pathways such as neuroinflammation, aberrant neuroplasticity, dysfunction of the MGB axis, and neuroendocrine abnormalities. These mechanisms interact with each other, forming a complex pathological network underlying PPD. Given the multifactorial pathophysiology of PPD, therapeutic strategies that target multiple pathways simultaneously—such as those employing botanical drugs and their metabolites—are generating increasing research interest. However, a systematic and critical evaluation integrating current knowledge of PPD pathogenesis with the evidence for the molecular mechanisms of TCM interventions is lacking. To provide a solid foundation for advancing fundamental research and clinical translation, this review aims to synthesize current knowledge on the pathogenic mechanisms of PPD and elucidate the pharmacological mechanisms by which TCM and natural products exert their effects against PPD.

## 2 Methodology

**FIGURE 1:**
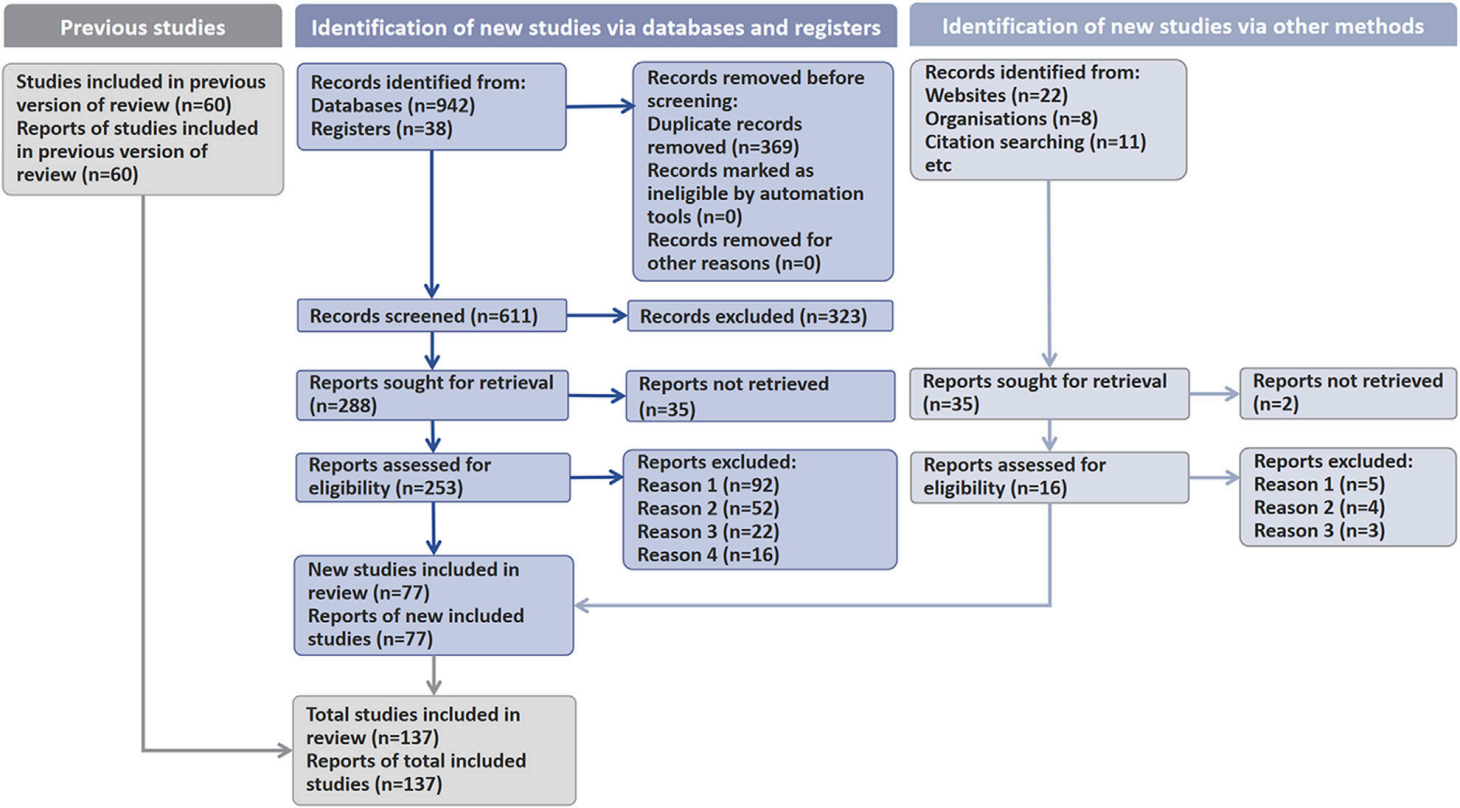
Flowchart of literature retrieval and screening.

This study followed the Preferred Reporting Items for Systematic Reviews and Meta-Analyses (PRISMA) guidelines and systematically searched for research on the pathological mechanisms, TCM interventions, PPD pharmacotherapies and their side effects. Data were sourced from PubMed, Web of Science, and ScienceDirect, with a search time range from January 2000 to the date the search was conducted (April 2024). The search strategy used a combination of subject headings and free-text terms, with English search terms including “postpartum depression”, “neuroinflammation”, “MGB axis”, “HPA axis”, “neuroplasticity”, “neuroendocrine”, “metabolites from traditional Chinese medicine”, “Traditional Chinese Medicine Formulas”, “natural products”, “toxicity”, and combined using Boolean operators (AND/OR). Inclusion criteria were: ① original research papers published in English; ② studies based on animal, cell model, or clinical trials of PPD; ③ studies describing the pathogenesis of postpartum depression (PPD); Exclusion criteria were: ① non-English literature; ② reviews, conference abstracts, case reports, and other gray literature; ③ duplicate publications; ④ studies lacking clear objectives, methodologies, or data related to PPD or traditional Chinese medicine or natural products interventions; ⑤ studies that did not provide sufficient experimental design or detailed data information. Literature screening was independently completed by two researchers, who first screened through titles and abstracts to exclude studies that clearly did not meet the criteria. Subsequently, the remaining literature was read in full text for further screening, and discrepancies were resolved through cross-checking. A total of 137 articles were finally included for systematic analysis (Figure 1).

## 3 Pathological mechanism of PPD

### 3.1 Neuroinflammation

The term neuroinflammation is now commonly used to refer to all immune activity within the central nervous system (CNS) in response to both acute and chronic diseases, including traumatic, infectious, ischemic, autoimmune, and degenerative conditions. Originally, it was used to describe the inflammatory response of microglia under pathological conditions (Nie et al., 2018). Accumulating evidence suggests a link between neuroinflammation and the pathophysiology of PPD. Neuroinflammation, which plays a dual role in exacerbating damage and contributing to repair, is considered crucial in the prognosis of many neurological diseases, including depression. One of the most common and serious disorders affecting the central nervous system is depression, and growing interest has been directed toward the link between depression and neuroinflammation. Numerous studies have shown that levels of inflammatory markers are elevated in individuals with depression. According to a meta-analysis, the clinical use of certain anti-inflammatory medications, such as statins, cytokine inhibitors, minocycline, and nonsteroidal anti-inflammatory drugs, has led to a significant reduction in depressive symptoms in some patients with severe depression (Köhler-Forsberg et al., 2019).

Research has shown that activation of the neuroimmune system during pregnancy-related stress significantly increases the levels of four pro-inflammatory substances in the serum of multiparous rats: interleukin-1α (IL-1α), interleukin-6 (IL-6), tumor necrosis factor-α (TNF-α), and interferon-γ (IFN-γ). This leads to a disruption of the neurotransmitter system, potentially contributing to the pathological development of inflammatory depression. Omega-3 polyunsaturated fatty acids (n-3 PUFAs) are crucial for human health. It is noteworthy that multiple studies in recent years have found that omega-3 polyunsaturated fatty acids can reduce pregnancy-related neuroinflammatory responses and exert antidepressant effects by inhibiting the production of pro-inflammatory cytokines and regulating neurotransmitter systems (Tang et al., 2018a). Further research has also found that, The CNS contains myeloid innate immune cells known as microglia, which have neuroprotective properties in both healthy and diseased states. Lipopolysaccharide (LPS) immune stimulation significantly activates microglia in the hippocampus of female mice, promoting the release of pro-inflammatory cytokines and inducing anxiety and depressive-like behavior. Notably, there is a significant positive correlation between the extent of microglia activation, the severity of depressive behavior, and the levels of neuroinflammatory markers. This suggests that neuroinflammatory pathways play a critical role in the development of depression. Furthermore, short postpartum separation (PS) during lactation can effectively inhibit excessive microglial activation, reduce the neuroinflammatory response, and significantly enhance the recovery of female mice from LPS-induced behavioral defects. These findings provide a theoretical basis for the prevention and treatment of PPD (Wu et al., 2021).

The cytoplasmic oligomer NOD-, LRR- and pyrin domain-containing protein 3 (NLRP3) is one of many inflammasome complexes, which are multiprotein signaling complexes. Inflammasomes are notably associated with autoimmune disorders and various self-inflammatory conditions in humans. Chronic stress can significantly activate the NLRP3 inflammasome pathway in the hippocampus, triggering neuroinflammatory responses and leading to an increase in postpartum depressive-like behavior. Exercise during pregnancy can regulate prolactin levels, which not only directly inhibits neuroinflammation but also exerts significant anti-inflammatory and neuroprotective effects on hippocampal tissue. This regulation helps prevent postpartum anxiety and depressive-like behaviors by systematically inhibiting hippocampal neuroinflammation, especially the NLRP3 inflammasome pathway. Moreover, prenatal stress significantly activates the NLRP3 inflammasome in the hippocampus, leading to the increased release of pro-inflammatory cytokines, cortical neuroinflammation, and neurodegeneration, ultimately resulting in offspring exhibiting depressive-like behavior. This clarifies how NLRP3 inflammasome activation plays a crucial role in the etiology of PPD (Özdamar et al., 2022). In summary, neuroinflammation plays a critical role in PPD, including abnormal activation of microglia, the release of NLRP3 inflammasomes, and the production of pro-inflammatory cytokines. Targeting neuroinflammation is a promising strategy for treating PPD.

### 3.2 MGB axis

The MGB axis has been implicated in various mood disorders, including PPD. This axis refers to the bidirectional communication between gut microbiota and brain function (Donald and Finlay, 2023). Despite the anatomical separation between the gut and brain, there is growing evidence that the gut microbiota and the CNS communicate bidirectionally (Ahmed et al., 2022). The MGB axis has been proposed as the communication link between gut bacteria and the CNS. Increasing research suggests that disturbances in gut microbiota composition in individuals with depression can influence both the pathological and physiological progression of the disorder in a variety of ways (Liu et al., 2023).

The pathophysiology of PPD is significantly influenced by the MGB axis. Studies have linked higher prenatal depression ratings to lower gut microbiome species richness, diversity, and evenness (Nel et al., 2025). Compared to healthy controls, PPD patients exhibit relatively low Firmicute abundance and notable variations in the presence of several key genera. Notably, the levels of certain genera, such as Phascolarctobacteria, Lachnospiraceae, *Faecalibacterium*, and *Tyzzerella*_3, are correlated with the severity of PPD (Zhou et al., 2020). The relationship between gut microbiota and PPD has also been investigated using the Mendelian randomization method, which further demonstrated that alterations in gut microbiota increase the risk of PPD (Sun et al., 2024). Animal studies have identified specific gut microbiota key to the development of PPD (Tian et al., 2021). Furthermore, antidepressant medications like fluoxetine (FLX) have been shown to exert anti-PPD effects by regulating the gut microbiota (Ramsteijn et al., 2020; Zheng et al., 2023). This further confirms the critical role of the MGB axis in the pathophysiological process of PPD.

The use of specific prebiotics and probiotics is generally safe and well tolerated. Research has shown that specific probiotic strains, such as *Lactobacillus rhamnosus*, can significantly reduce the incidence of PPD and anxiety in pregnant and postpartum women, while being safe and tolerable for both mother and baby (Slykerman et al., 2017). *Lactobacillus casei* may alleviate PPD by modulating the brain-derived neurotrophic factor (BDNF)-extracellular signal-regulated kinase 1/2 (ERK-1/2) pathway, gut microbiota composition, brain monoamine levels, and oxidative stress (Yang et al., 2022). Furthermore, *L. rhamnosus* has been shown to enhance mother-infant bonding affected by PPD, reduce prenatal psychological burden, and improve maternal mental health (Benlakehal et al., 2025). Some studies suggest that anti-inflammatory medications could prevent and treat depression by altering the composition and quantity of beneficial gut bacteria, highlighting the potential of targeting gut microbiota and the NLRP3 inflammasome as strategies for preventing PPD. The NLRP3 inflammasome plays a crucial role in the MGB axis (Getachew et al., 2018; Song et al., 2022; Liu et al., 2020). Additionally, postpartum obesity increases the risk of PPD, and high dietary fiber intake has been identified as a promising nutritional strategy to prevent anxiety behaviors associated with prenatal obesity through the MGB axis (Liu et al., 2020). Research also suggests that malfunction of neuroplasticity and inflammasomes may be crucial in mediating the link between stress and gut microbiota composition (Zhou et al., 2023). Beyond posing a threat to maternal physical and mental health, PPD has been linked to negative effects on infant behavior and development. Studies indicate that maternal PPD may influence the neurological development of offspring by altering the composition of infant fecal microbiota and metabolites (Zhou et al., 2024). Therefore, identifying key gut microbiota strains and their functional metabolites related to neurodevelopment is essential for developing new treatment strategies for both mothers and infants affected by PPD.

In conclusion, PPD is strongly associated with alterations in particular bacterial genera and the overall composition of gut microbiota via the MGB axis. A promising direction for future research lies in the prevention and treatment of PPD through targeted microbiota modulation and the use of specialized probiotic interventions.

### 3.3 Neural plasticity

A fundamental concept in neuroscience, neuroplasticity refers to the brain’s ability to form new neural connections in response to learning, adaptation, and injury. This flexibility is essential for cognitive processes such as memory and learning, as well as for recovery after brain damage. Neuroplasticity can be divided into two main categories: structural plasticity and functional plasticity. Structural plasticity involves changes in neurogenesis, dendritic spine formation, and axonal growth and repair mechanisms. Functional plasticity, on the other hand, modifies the interactions between neurotransmitters and receptors, allowing the brain to adapt and maintain function without changing its physical structure (Chen S. et al., 2024).

The development and incidence of PPD are closely linked to neuroplasticity. Ketamine, a noncompetitive N-methyl-D-aspartate receptor (NMDAR) antagonist, has shown significant and rapid antidepressant effects (within hours) when used to treat refractory depression (Smith-Apeldoorn et al., 2022). Research indicates that S-ketamine (esketamine), the S-enantiomer of ketamine, enhances synaptic function by increasing synaptic protein levels and transmission efficiency, helping to combat depression. Notably, low-dose S-ketamine may have a unique dual effect of both antidepressant and anti-anxiety properties, while high-dose S-ketamine does not exhibit this effect (Ren et al., 2022). Anxiety and depression are heavily influenced by disruptions in the excitatory/inhibitory (E/I) ratio in the cortex, leading to abnormal functional connectivity and synaptic alterations in excitatory and inhibitory neurotransmitters (Hartmann et al., 2017; Lener et al., 2017; Pham and Gardier, 2019). Administering classic Wnt signaling agonists to restore Wnt signaling can help rebalance the E/I ratio and alleviate depression-related symptoms by enhancing the plasticity of gamma-aminobutyric acid (GABA) and dopaminergic synapses (Ye B. et al., 2023).

Recent studies have shown that the functional imbalance of the GABAergic system and neurosteroid withdrawal play a key role in the pathogenesis of PPD. Allopregnanolone (ALLO) is the main metabolite of progesterone, which can act as a positive allosteric modulator of GABA_A_ receptors, enhancing inhibitory transmission and maintaining emotional stability (Wang, 2011). Clinical observations have found that the level of ALLO increases significantly during pregnancy and decreases rapidly after childbirth, triggering the so-called neurosteroid withdrawal syndrome (Maguire and Mody, 2008). This hormonal shift leads to the restructuring of GABA_A_ receptor subunits and a reduction in inhibitory control, resulting in emotional imbalance (Maguire and Mody, 2008; Smith et al., 2007). Clinical studies further confirm that reduced ALLO levels in the third trimester of pregnancy are significantly associated with depressive symptoms (Hellgren et al., 2014). Furthermore, the intravenous agent Brexanolone (an ALLO derivative) has demonstrated rapid and sustained antidepressant efficacy in patients with PPD, further supporting the GABAA receptor–neurosteroid withdrawal hypothesis (Me et al., 2018).

The hippocampus of rodents produces new neurons throughout their lifespan, a process known as adult hippocampal neurogenesis (AHN) (Moreno-Jiménez et al., 2021). As a unique form of neural circuit plasticity, AHN is fundamental to cognitive and emotional regulation. Short PS can activate AHN in female mice, enhancing their cognition and improving resistance to PPD-like behavior (Zhou et al., 2025). The novel σ-1 receptor agonist YL-0919 activates the σ-1 receptor, regulates GABA/glutamate signaling, restores the balance of excitation and inhibition in the brain, and promotes hippocampal plasticity, providing insight for the development of new PPD treatments (Ren et al., 2024). Repetitive transcranial magnetic stimulation (rTMS) is a non-invasive technique that uses magnetic fields to regulate brain regions associated with depression (Garcia et al., 2010). Studies have shown that rTMS combined with oxytocin (OT) can enhance the alleviation of PPD-like behavior through mechanisms such as glial cell activity regulation and synaptic plasticity enhancement (Amin et al., 2025). In addition, music therapy may prevent PPD by protecting synaptic plasticity from oxidative stress and inflammatory damage caused by estrogen imbalance (Fu et al., 2025a). Environmental enrichment (EE) has been found to effectively reduce neuronal inflammation, apoptosis, and synaptic plasticity damage, thereby alleviating PPD-like behavior induced by maternal-infant separation in female mice (Chen G. et al., 2024). Overall, the pathophysiology and treatment of PPD are significantly influenced by neuroplasticity, including neurotransmitters, synaptic plasticity, and neurogenesis. Controlling neuroplasticity represents a key strategy for PPD treatment.

### 3.4 Neuroendocrine

The neuroendocrine system, which regulates and balances hormone secretion and function, serves as the connection between the endocrine glands and the CNS (Datta et al., 2023). An essential component of this system, the hypothalamic-pituitary-adrenal (HPA) axis, plays a critical role in controlling the body’s stress response and various other physiological functions (Zheng et al., 2024).

During pregnancy and the postpartum period, the maternal hypothalamic-pituitary-adrenal (HPA) axis functions differently, and these changes may be closely linked to the development of PPD. Research has shown that postpartum women with depression exhibit reduced HPA reactivity and weakened circadian rhythm changes in cortisol levels compared to women without depression (De Rezende et al., 2018). Additionally, plasma or serum levels of docosahexaenoic acid (DHA) in women with PPD are significantly lower than in asymptomatic individuals. Studies have found that the reduction of DHA, particularly in women with multiple pregnancies, may be associated with altered expression of glucocorticoid receptors (GR), which exacerbates depressive-like behavior and activates the HPA axis (Tang et al., 2018b). GR, a crucial signaling molecule of the HPA axis, is linked to anxiety and depressive-like behaviors and is downregulated in the hippocampus during postpartum withdrawal symptoms (Wang et al., 2020). Furthermore, PPD appears to result from both prenatal stress and inhibition of postpartum HPA axis activity (HPA-AA). Studies suggest that hair steroid analysis may be used to predict the risk of PPD (Sadeghi-Bahmani et al., 2025). Multiple stressors have been shown to induce PPD by affecting the HPA axis. Chronic psychological stress during pregnancy, for example, can disrupt adaptive changes in the maternal neuroendocrine system by altering HPA axis activity (Zoubovsky et al., 2020). Stress, along with fluctuations in ovarian hormones, can also contribute to PPD by disrupting HPA axis components, such as corticotropin-releasing factor, cortisol, and GR (Payne and Maguire, 2019). Vasopressin, which is vital to the stress response (Seth et al., 2016), has also been implicated in PPD, as it may promote dysfunction of the HPA axis (Kashkouli et al., 2023). However, it is important to note that long-term HPA axis dysfunction, as measured by hair cortisol concentration at 12 months postpartum, does not mediate the substantial increase in PPD risk and symptoms associated with childhood maltreatment (Blecker et al., 2025).

HPA axis dysfunction interacts with other pathological processes, collectively contributing to the development of PPD and related complications. *Postartum* female mice exposed to low-resource environments may experience a lack of pleasure, elevated cortisol levels, and neuroinflammation. These changes can lead to the development of PPD and postpartum hypertension, which are driven by elevated corticosterone levels and neuroinflammation (Jones et al., 2025). The mechanisms through which poverty exacerbates depression are believed to include HPA axis dysregulation, neuroinflammation, and dietary changes, which result in excessive circulating lipids and blood sugar (Osborne et al., 2020; Lloyd-Jones et al., 2022). Studies have shown that mice treated with high levels of postpartum corticosterone (CORT) exhibit HPA axis activation, depressive-like behavior, and impaired maternal care. Notably, maternal CORT-induced PPD can also impair offspring HPA axis function (Xie et al., 2025). In terms of intervention, FLX, the most commonly used prescription medication for maternal depression, combined with EE, has been shown to significantly increase OT levels in the brain. This combination helps alleviate anxiety and depressive behaviors by reducing HPA axis dysfunction in mothers following stress exposure (Bashiri et al., 2021). Additionally, n-3 polyunsaturated fatty acids (PUFAs) may improve PPD by modifying HPA axis function, reducing neuroinflammation, and regulating related miRNAs through the 5-hydroxytryptamine pathway (Choi et al., 2020). In summary, the dynamic changes and dysregulation of the postpartum HPA axis form a core pathological mechanism of PPD by affecting key molecules such as GR, mediating various stress responses, and interacting with other pathological processes.

### 3.5 Other aspects

The occurrence and development of PPD are influenced by various factors, making in-depth research into its mechanisms, prediction, and treatment strategies crucial. Studies have shown that aberrant signaling in the central amygdala, involving lipid transport protein type prostaglandin D synthase (L-PGDS)/prostaglandin D2 (PGD2), disrupts Src protein phosphorylation, leading to astrocyte atrophy and ultimately resulting in PPD in mice (Sheng et al., 2024). Pathway analysis also suggests that DNA methylation patterns associated with hippocampal synaptic plasticity could play a key role in the onset of PPD (Guintivano et al., 2014). A meta-analysis of genome-wide association studies identified genetic links between PPD and major depression, bipolar disorder, anxiety, PTSD, insomnia, menarche age, and polycystic ovary syndrome. Notably, cell enrichment analysis revealed that the only FDA-approved medication for PPD shares a mechanism of action with GABAergic neurons (Guintivano et al., 2023). Furthermore, postpartum mice subjected to environmental stressors, such as reduced nighttime light exposure, may exhibit depressive-like behavior. This effect is likely mediated by disruptions in circadian rhythms and gene expression patterns (Lin et al., 2025). Changes in the levels of specific metabolites, including decanoic acid, dodecanoic acid, arachidic acid, behenic acid, and L-tryptophan, found in cerebrospinal fluid, show strong correlations with PPD symptoms. Due to their high discriminatory performance, these metabolites have potential as predictive biomarkers for PPD (Sheng et al., 2023). Moreover, another study suggests that assessing thyroid function, inflammatory markers (such as ferritin and C-reactive protein), pregnancy-related hormones (such as estrogen and progesterone), vitamin D levels, and coagulation indicators, in addition to clinical evaluations, may help identify individuals at high risk for perinatal depression early on (Ciolac et al., 2025).

**FIGURE 2:**
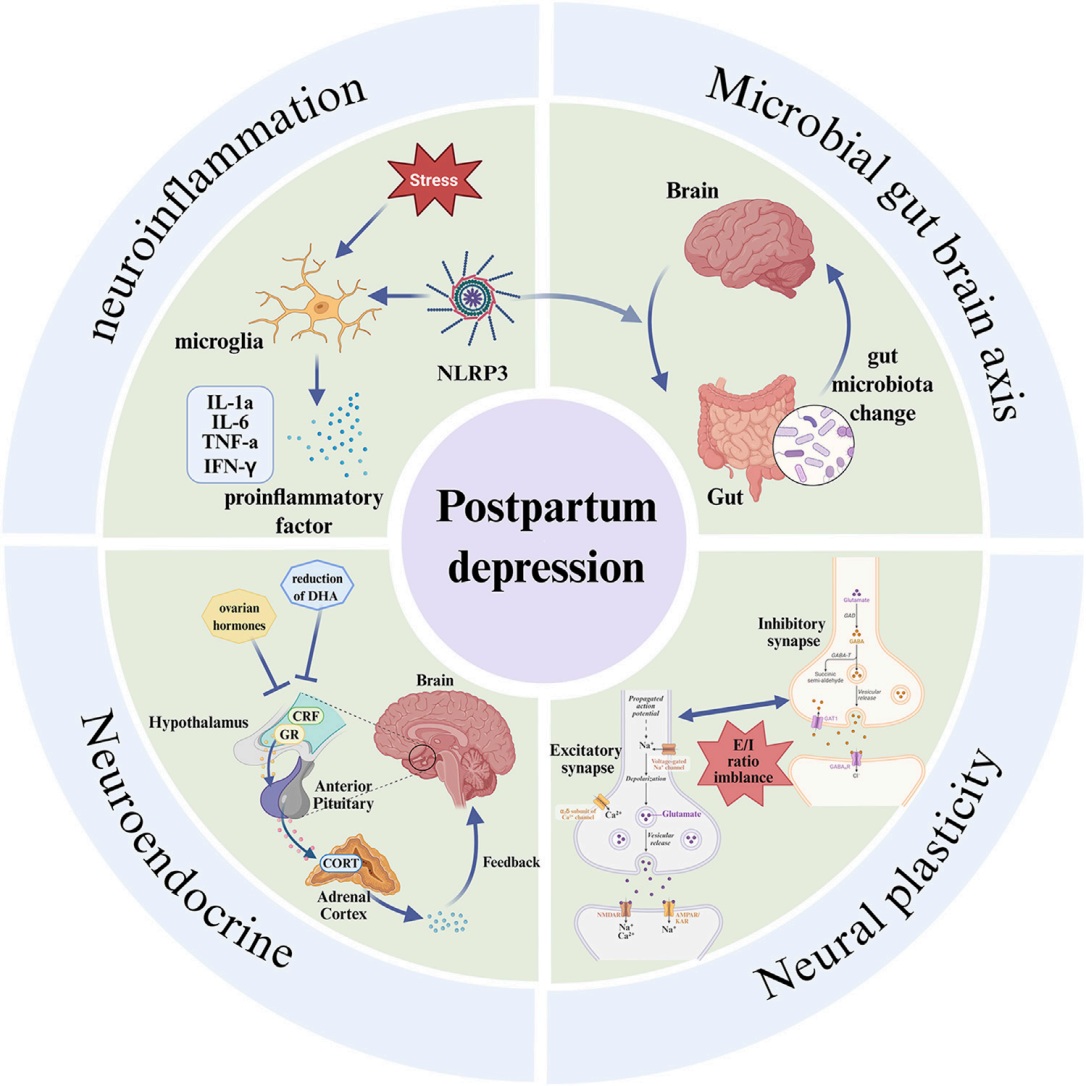
Pathological mechanisms of PPD. The pathological mechanisms of PPD involve neuroinflammation, microbial gut brain axis, neural plasticity, neuroendocrine. When the relevant pathological mechanisms are impaired or affected, they respond through pathways that lead to the development of PPD.

Research has suggested that hippocampal CA3 signaling protein 3A (sema3A) may be a potential therapeutic target and plays a crucial role in the pathophysiology of PPD (Chen et al., 2025). Furthermore, studies have shown that PHD finger-containing protein 1 (BRPF1) and the bromodomain of lysine demethylase 3A (KDM3A) are important regulatory factors for iron toxicity sensitivity in PPD. This molecular axis is primarily regulated by the transcription of glutathione peroxidase 4 (GPX4) and the cysteine/glutamate antiporter SLC7A11, which help maintain redox homeostasis. Disruption of this pathway can trigger a neuropathic cascade reaction. Therefore, drug-targeted interventions within these pathways offer a promising neuroprotective strategy for PPD treatment (Lan et al., 2025). In summary, the pathogenesis of PPD involves multiple signaling pathways and complex molecular interactions, many of which remain poorly understood. To lay a solid foundation for developing more effective preventive and therapeutic strategies, it is essential for future studies to continue exploring and methodically analyzing the physiological and pathological mechanisms underlying PPD. Identifying key regulatory nodes and potential intervention targets will be critical for advancing both basic research and clinical application (Figure 2; Table 1).

**TABLE 1:** Pathological mechanism information of PPD.

Modeling method	Involving behavioral test	Main mechanism of action	References
Gestational stress	FST, SPT	Supplementing with Omega-3 during pregnancy can alleviate neuroinflammation and regulate neurochemistry in rats	Tang et al. (2018a)
Prenatal stress, postpartum LPS	OFT, FST	Transient PS enhances the resistance of female mice to LPS induced depression like and anxiety like behaviors, achieving neuroprotective effects by inhibiting hippocampal microglial activation and neuroinflammation	Wu et al. (2021)
Chronic unpredictable mild stress	OFT, SPT	Prenatal stress may lead to depression like behavior in offspring by activating hippocampal NLRP3 inflammasome and cortical neuropathy	Özdamar et al. (2022)
Maternal separation	SPT, EPM, FST, TST	*Lactobacillus* casei can improve postpartum depression by altering the composition of the gut microbiota, monoamine substances in the brain, and oxidative stress	Yang et al. (2022)
Prenatal restraint stress	Maternal care (licking/nursing), locomotor reactivity, EPM	*Lactobacillus* reuteri restores gut microbiota balance, improves CORT/OT levels and hypothalamic OT-BDNF axis function, thereby promoting maternal behavior recovery and emotional stability	Benlakehal et al. (2025)
Hormone/estrogen withdrawal model	SPT, FST, TST, OFT	Estrogen withdrawal may lead to depressive like behavior through dysbiosis of the gut microbiota and activation of NLRP3 inflammasome in the hippocampus	Xu et al. (2024)
Chronic unpredictable mild stress	OFT, SPT, FST	CUMS may lead to depressive like behavior through dysbiosis of gut microbiota and neuroinflammatory response (NF - κB signaling pathway); Puerarin can improve depressive like behavior by regulating gut microbiota	Song et al. (2022)
Antenatal high-fat diet	MWM, TST, OFT, EPM	High dietary fiber intake may alleviate postpartum depression like behavior by promoting SCFAs generation, reshaping gut microbiota, regulating 5-HT and NE, reducing inflammation, and improving intestinal barrier function	Liu et al. (2020)
Chronic restraint stress, long pup separation	OFT, EPM, TST, FST, SPT	Long term separation combined with chronic stress may induce postpartum depression like behavior by activating microglia, upregulating the NLRP3 inflammatory pathway, and reducing neuroplasticity related proteins	Zhou et al. (2023)
Hormone withdrawal	SPT, Locomotor activity, EPM, FST	S-ketamine exerts antidepressant effects by increasing the number of synaptic connections and synaptic transmission efficiency in postpartum depressed rats	Ren et al. (2022)
Conditional glucocorticoid receptor knockout mice (GRGlu-CKO, GRGABA-CKO)	FST, OFT, fear conditioning, chronic social defeat stress	The absence of glutamatergic neurons GR in the forebrain leads to impaired negative feedback regulation of the HPA axis, upregulation of CRH mRNA, and elevated levels of corticosterone, causing anxiety and fear related behavioral abnormalities	Hartmann et al. (2017)
Bilateral ovariectomy, Hormone-simulated postpartum period	OFT, SPT	Restoring Wnt signaling helps alleviate depressive like behavior by enhancing the plasticity of inhibitory and excitatory synapses in the hippocampus and rebalancing the excitation/inhibition ratio	Ye et al. (2023a)
GABA_A receptor δ-subunit deficient mice (Gabrd+/−, Gabrd−/−)	Depression-like behavior tests, anxiety-like behavior tests, maternal behavior observation, pup survival analysis	*Postartum* neurosteroid hormone levels decrease, and GABAA receptor delta subunit defects lead to the failure of tetanic GABAergic inhibition regulation, making it difficult to adapt to postpartum hormone changes and causing postpartum depression like behavior	Maguire and Mody (2008)
Pup Sepamiceion, Chronic Restraint Stress	NORT, PRT, OFT, EPM, TST	Adult hippocampal neurogenesis mediated cognitive behavior is involved in the stress recovery of anxiety and depression like behaviors in postpartum female mice after CRS.	Zhou et al. (2025)
Ovarian hormone withdrawal	OFT, EPM, SPT, TST, FST	YL-0919 activates the σ −1 receptor, restores GABA/Glu balance, regulates HPA axis activity, activates the BDNF/p-mTOR signaling pathway, promotes hippocampal neural plasticity, and rapidly improves PPD like anxiety and depressive behavior	Ren et al. (2024)
Maternal Separation with Early Weaning	Coat test, Splash test, SPT, FST, TST	The combination therapy of rTMS and oxytocin may improve postpartum depression behavior by downregulating cell apoptosis, increasing synaptic plasticity, regulating the activity of microglia and astrocytes, and regulating autophagy	Amin et al. (2025)
Ovarian hormone withdrawal	OFT, FST, EPM, SPT, NSF, TST	Music therapy prevents PPD by regulating oxidative stress, inflammation, and synaptic integrity	Fu et al. (2025a)
Maternal Separation	OFT, EPM, FST, SPT, Barnes maze test, New object recognition	Environmental Enrichment significantly improves MS induced PPD like behavior, inhibits neuroinflammation and neuronal apoptosis, enhances synaptic plasticity and neurogenesis, thereby reversing depression and cognitive impairment	Chen et al. (2024b)
Hormone-stimulated pregnancy with estradiol withdrawal	OFT, EPM, FST, TST	Estrogen withdrawal may lead to PPD like behavior by inhibiting hippocampal glucocorticoid receptor expression	Wang et al. (2020)
Chronic psychosocial stress during pregnancy	Pup retrieval, FST, SPT, EZM	Chronic psychosocial stress may lead to PPD like behavior by interfering with perinatal HPA axis adaptation and increasing PVN CRH signaling	Zoubovsky et al. (2020)
*Postartum* corticosterone treatment	SPT, TST, FST, OFT, MWM	Corticosteroids may affect the BDNF mTOR signaling pathway in the hippocampus by activating the HPA axis, leading to neuronal damage and depressive like behavior	Xie et al. (2025)
Hormone-simulated pregnancy, pup separation	FST, SPT, maternal behavior tests	N-3PUFA improves PPD through the 5-hydroxytryptamine pathway by altering the HPA axis, neuroinflammation, and related miRNAs	Choi et al. (2020)
Dim light at night exposure model (dLAN)	SPT, FST, OFT, maternal behavior monitoring	Nighttime dim light may disrupt circadian rhythm behavior and gene expression, affecting 5-HT and BDNF levels, leading to PPD	Lin et al. (2025)
Ovarian hormone withdrawal model	FST, TST	*Postartum* estrogen withdrawal may lead to depression like behavior by upregulating semaphorin 3A expression in the hippocampus, inhibiting dendritic complexity and synaptic plasticity of hippocampal neurons	Chen et al. (2025)

## 4 Molecular mechanisms of natural products

Several medicinal plants and their characterized metabolites, such as those from *Hypericum perforatum* L. (Hypericaceae) have been investigated or applied in the context of PPD management. Among its characterized metabolites, hypericin is particularly important for its pharmacological activity (Butterweck and Schmidt, 2007; Zhai et al., 2015; Grundmann et al., 2006). Studies suggest that the metabolite hypericin may alleviate PPD symptoms by modulating glucocorticoid metabolism, upregulating estrogen receptor expression, and reducing neuroinflammation (Zhai et al., 2022). Existing reviews and clinical reports indicate that the overall tolerance is good, but mild gastrointestinal discomfort and photosensitivity may occur (Brockmöller et al., 1997; Woelk et al., 1994), individual cases have manic episodes (Burt et al., 1999). The use of *H. perforatum* L. (Hypericaceae) in early pregnancy has not been shown to significantly increase the risk of premature birth, stillbirth, or major malformations (Moretti et al., 2009), but some studies suggest that the incidence of neonatal malformations may be slightly higher than in the general population (Kolding et al., 2015). Overall, the evidence suggests safety, but further high-quality research is needed to validate its long-term safety and interactions (Rodríguez-Landa and Contreras, 2003; Zepeda et al., 2023). Total Glycosides of Paeonia, a standardized extract from the roots of *Paeonia lactiflora* Pall. (Paeoniaceae), contains paeoniflorin as its primary metabolite (Zhang and Wei, 2020). Paeoniflorin, as a key glycoside in Paeonia plants, has garnered attention for its potential neurotrophic effects (Li et al., 2017). BDNF, which plays a critical role in regulating emotional disorders, is notably influenced by Paeoniflorin. Imbalanced levels of BDNF are strongly linked to depression, and paeoniflorin may improve PPD by modulating BDNF and activating the mTOR signaling pathway (Erickson et al., 2012; Haile et al., 2014). In addition, paeoniflorin has shown positive effects on Translocator Protein (TSPO), an 18 kDa protein with known anti-anxiety and anti-depressant properties (Chen et al., 2022; Rupprecht et al., 2009; Costa et al., 2012). Toxicity studies indicate that paeoniflorin has low acute toxicity, minimal subacute and chronic toxicity, and no genetic or mutagenic effects (Ou et al., 2025). However, it is important to note that paeoniflorin and its metabolites are primarily excreted via the kidneys (Shao et al., 2017). Therefore, patients with impaired kidney function may require dosage adjustments to avoid medication accumulation and potential toxicity. Another promising metabolite is timosaponin B-Ⅲ, a steroidal saponin isolated from the rhizomes of *Anemarrhena asphodeloides* Bunge (Asparagaceae). In PPD mouse models, TB-Ⅲ has exhibited antidepressant effects. Its mechanisms of action are believed to involve the regulation of inflammatory cytokines, BDNF signaling, and synaptic plasticity (Zhang et al., 2017).

Stevioside, a key diterpenoid metabolite derived from the leaves of *Stevia rebaudiana* (Bertoni) Bertoni (Asteraceae), is widely known as a calorie-free sweetener due to its potent sweetness (250–300 times sweeter than sucrose) (Chatsudthipong and Muanprasat, 2009). Recent studies suggest that gestational obesity, induced by a high-fat diet during pregnancy, is positively correlated with depression, anxiety, and cognitive impairment in pregnant rats (Liu et al., 2020). Interestingly, stevioside has been shown to reverse PPD-like behavior and cognitive impairments caused by high-fat diets. The mechanism of action involves repairing intestinal barrier structure, reducing serum LPS levels, inhibiting microglial activation, and reducing the release of pro-inflammatory cytokines. Furthermore, stevioside enhances brain antioxidant capacity and improves mitochondrial function, offering a promising therapeutic approach for PPD (Ye et al., 2024). Another notable metabolite in the realm of TCM is Epigallocatechin-3-gallate (EGCG), a catechin polyphenol found in *Camellia sinensis* (L.) Kuntze, Theaceae. Known for its anti-inflammatory, antioxidant, and neuroprotective properties, EGCG has shown potential as a therapeutic agent for various illnesses, including mental health disorders (Chakrawarti et al., 2016). Research indicates that Sema3A, a molecule elevated in certain mental illnesses (Van Battum et al., 2015), is associated with severe depression (Zhou et al., 2017). As a downstream target of Sema3A, glycogen synthase kinase 3β (GSK3β) is thought to play a role in depression pathology. Studies suggest that EGCG can reduce depressive-like behaviors in mice by increasing the phosphorylation levels of GSK3β and decreasing Sema3A expression in the hippocampus. This indicates that EGCG may hold promise as a potential treatment for PPD, especially by targeting the neuroinflammatory and synaptic plasticity pathways involved in depression (Xu et al., 2023).

Curcumin, the primary curcuminoid metabolite of *Curcuma longa* L. (Zingiberaceae) (a member of the ginger family), has been used as both a culinary spice and a medicinal plant in South Asia for thousands of years (Kunnumakkara et al., 2017; Farzaei et al., 2018). Clinical trials have shown that curcumin can effectively reduce postpartum anxiety and depression. Its mechanism of action involves synergistic effects across multiple pathways, including neurotransmitter regulation, HPA axis modulation, inflammation inhibition, antioxidant activity, and promotion of neural nutrition (Hamlbar et al., 2025). Curcumin is non-mutagenic and non-genotoxic, making it a relatively safe option. However, some studies indicate that curcumin may be poorly absorbed in the gastrointestinal system. Oral administration of 6g/day has been deemed safe for 4–7 weeks, although gastrointestinal discomfort can occur in some individuals (Soleimani et al., 2018). Resveratrol (RSV), a polyphenolic metabolite found in red grape skins (*Vitis vinifera* L., Vitaceae), red wine, and peanuts, is an activator of Silent Information Regulator 1 (Sirt1), which plays a key role in processes like oxidative stress, glucose and lipid metabolism, and cell cycle regulation (Manetti et al., 2022). Research indicates that RSV (Wang et al., 2018) can reduce PPD-like behavior in mice by blocking the AKT/mTOR signaling pathway, promoting SIRT1 activation, and inducing autophagy (Ye S. et al., 2023). However, RSV has low solubility and poor bioavailability, which significantly limits its effectiveness *in vivo* (Walle et al., 2004). Moreover, the safety profile of resveratrol requires careful evaluation. Studies suggest that its effects are not only dose-dependent but also involve complex hormone-modulating activities, while high doses can be toxic (Calabrese et al., 2010). Therefore, comprehensive preclinical studies are imperative to establish a safe therapeutic window, especially for the vulnerable postpartum population. In summary, while both curcumin and RSV show promise in alleviating PPD symptoms, more research is required to optimize their use and ensure safety, especially with regard to their bioavailability, toxicity, and molecular mechanisms.

## 5 Molecular mechanisms of traditional Chinese medicine formulas

With its long history of treating various ailments, TCM constitutes a prime example of ethnopharmacology. Ethnopharmacology, the interdisciplinary study of traditionally used medicinal substances, provides a key framework for understanding TCM (Assadi et al., 2011). With its rich cultural heritage, TCM employs a diverse arsenal of plant-based remedies, minerals, and animal products to treat a wide range of diseases, including PPD. Consequently, the application of TCM in modern therapy, particularly for PPD, is gaining increasing attention as it may offer novel, multi-target mechanisms derived from these traditional practices.

Sugemule-7 is a TCM formulation composed of several botanical drugs, including Nutmeg (*Myristica fatua* Houtt. (Myristicaceae)), Amomum cardamomum L. (Zingiberaceae), Clove (*Syzygium aromaticum* (L.) Merr. and L.M. *Perry (*Myrtaceae)), Asparagus (*Asparagus cochinchinensis* (Lour.) Merr. (Asparagaceae)), *Gymnadenia conopsea* (L.). R. Br. (Orchidaceae), *Aquilaria agallocha* Roxb. (Thymelaeaceae), Siberian solomonseal rhizome (*Polygonatum sibiricum* Redouté (Asparagaceae)) (Shi et al., 2023). Research on its constituent botanical drugs, such as Myristica fragrans (nutmeg), has identified metabolites with neuroprotective, antioxidant, and anti-inflammatory properties (Barman et al., 2021; Valderrama, 2000). Preclinical studies suggest that the formulation Sugemule-7 might have potential in alleviating PPD, as indicated by mechanisms involving neuroprotection, reduction of oxidative stress and neuroinflammation, and enhancement of synaptic plasticity (Fu et al., 2025b).

Shen-Qi-Jie-Yu-Fang (SJF) is a TCM formulation composed of the following eight botanical drugs: *Astragalus membranaceus* (Fisch.) Bunge (Fabaceae), *Codonopsis pilosula* (Franch.) Nannf. (Campanulaceae), *Ziziphus jujuba* Mill. var. *spinosa* (Bunge) Hu ex H.F. Chow (Rhamnaceae), *Cornus officinalis* Siebold & Zucc. (Cornaceae), *Curcuma zedoaria* (Christm.) Roscoe (Zingiberaceae), *Citrus reticulata* Blanco (Rutaceae), *Citrus medica* L. (Rutaceae), *Angelica sinensis* (Oliv.) Diels (Apiaceae). Activation of the T cell system has been linked to the susceptibility of emotional disorders (Drexhage et al., 2011). CD4^+^CD25^+^ regulatory T (Treg) cells, a subset of CD4^+^ T-helper cells, play a key role in inhibiting excessive T cell responses. Research indicates functional impairments between pro-inflammatory cytokines and regulatory T cells (Tregs) at various stages of PPD. Studies show that SJF and FLX may mitigate this functional impairment by controlling the expression of CD4^+^CD25^+^ Treg cells, gp130, IL-1RI, and the serum concentrations of IL-1β and IL-6 (Li et al., 2016). Moreover, soluble epoxide hydrolase (sEH) inhibitors can alleviate inflammatory responses by increasing the levels of Epoxyeicosatrienoic acids (EETs), thereby rapidly improving the depressive state of mice (Ren et al., 2016). By blocking the inflammatory signaling system in the hippocampal tissue of PPD rats specifically the arachidonic acid metabolic pathway and nuclear factor kappa-B (NF-κB), SJF appears to have an effect similar to that of sEH inhibitors, helping to reduce the symptoms of PPD (Jingya et al., 2024).

**FIGURE 3:**
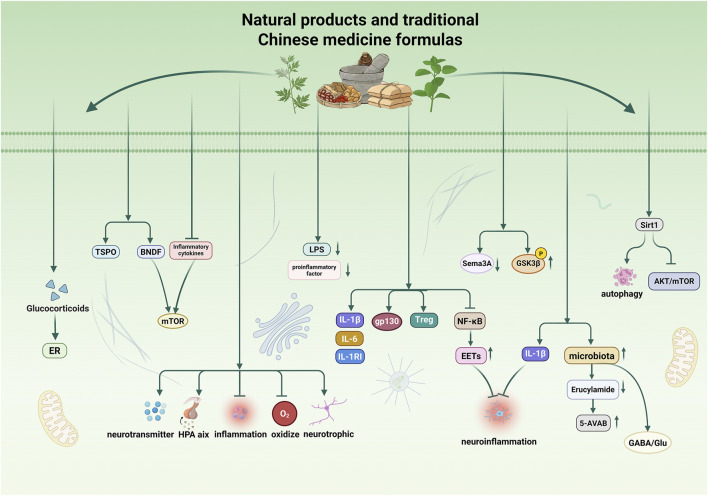
Mechanisms of action of Natural products and traditional Chinese medicine formulas. TCM can exert antidepressant effects in PPD through relevant targets and signalling pathways, including enhancing neuroplasticity, alleviating neuroinflammation, modulating monoamine neurotransmitters, regulating oxidative stress, stabilizing the HPA axis, protecting the blood-brain barrier, and maintaining the gut-brain axis balance. These actions further contribute to the treatment of PPD by relieving symptoms via appropriate pathological mechanisms.

919 Granules is a nationally patented TCM formulation consisting of the following botanical drugs: *Actinidia chinensis* Planch. (Actinidiaceae), *Salvia miltiorrhiza* Bunge (Lamiaceae), *Atractylodes macrocephala* Koidz. (Asteraceae), *Epimedium brevicornu* Maxim. (Berberidaceae), *Wolfiporia cocos* (F.A. Wolf) Ryvarden & Gilb. (Polyporaceae), *Bupleurum chinense* DC. (Apiaceae), *Schisandra chinensis* (Turcz.) Baill. (Schisandraceae), *Alpinia katsumadai* Hayata (Zingiberaceae), *C. reticulata* (pericarp), and *Pseudostellaria heterophylla (*Miq.) Pax (Caryophyllaceae). Research into the mechanisms of 919 Granules has explored its potential interaction with various endogenous signaling pathways. For example, it is hypothesized that the formulation may influence the activity of anandamide, a fatty acid amide that, through its receptors, regulates processes such as angiogenesis (Bezuglov et al., 1998) and water homeostasis (Hamberger and Stenhagen, 2003). Another molecule of interest is 5-AVAB, a trimethyl molecule essential for the growth and development of the embryonic nervous system (Haikonen et al., 2022). Research suggests that 919 Syrup may help alleviate PPD by boosting 5-AVAB, reducing erucamide levels in the hippocampal region of mice, and increasing the diversity of gut bacteria, including Bacteroidetes and *Lactobacillus* (Zheng et al., 2023). Another study indicates that 919 Syrup regulates gut flora and modifies metabolites to enhance hippocampal GABA/glutamate system activity, thus reducing PPD symptoms (Tian et al., 2021). However, an excess of pro-inflammatory cytokines in peripheral blood can damage the blood-brain barrier (BBB) by triggering astrocyte activation and decreasing transendothelial resistance. This allows peripheral immune cells or cytokines to enter the brain, causing inflammation, which can, in turn, contribute to depression. Excessive levels of IL-1β have been shown to damage the BBB (Hauptmann et al., 2020; Argaw et al., 2006; Hu et al., 2022). 919 Granules have been shown to reduce PPD symptoms by controlling IL-1β levels (Wang et al., 2024). In summary, TCM formulas such as Sugemule-7, SJF, and 919 Granules have shown efficacy in alleviating PPD, and their effects involve various molecular mechanisms. However, further clarification is needed regarding the specific metabolites of these compounds, including identifying the key active metabolites and determining which metabolites can cross the BBB to exert therapeutic effects on the CNS (Figure 3; table 2).

**TABLE 2:** Information on the effects of natural products and TCM formulas.

Natural products/TCM formulas	Source/Composition	Study subjects	Modeling method	Dosage and duration	Main indicators	Mechanism of action	References
Hypericin	*Hypericum perforatum*	Animals (Female SD rats, PPD model)	hormone-simulated pregnancy model	6.12 mg/kg, 1.0 mL/100 g body weight, once daily (Day 36–77, 42 days) Total experiment duration: 77 days	Open field test, Sucrose preference test, Body weight, Food intake, Adrenal/thymus/spleen indices	Relieve glucocorticoid metabolism, increase ER expression, and thereby alleviate neuroinflammation	Zhai et al. (2022)
Paeoniflorin	Paeonia lactiflora Pallas	Animals (Female SD rats, PPD model)	hormone-simulated pregnancy	20 mg/kg	SPT, FST, TST, E2 and P (Radioimmunoassay), Cor, Allo, IL-1β, TNF-α (ELISA), ERα and ERβ (qPCR), TSPO, BDNF, mTOR (Western blotting)	Activate TSPO and BDNF-mTOR pathways	Chen et al. (2022)
Timosaponin B-III	Anemarrhena asphodeloides Bge	Animals (Female mice, PPD model)	the administration of dexamethasone sodium phosphate during pregnancy	10, 20, 40 mg/kg, orally, once a day for 7 days	FST, TST, Inflammatory cytokines (serum and hippocampal tissue, ELISA), BDNF, GSK-3β, GluR1, PSD95, Synapsin I (Western blotting)	Regulate inflammatory cytokines, activate BDNF signaling pathway, and modulate synaptic plasticity-related proteins	Zhang et al. (2017)
Stevioside	Stevia rebaudiana leaf	Animals (Female C57BL/6J mice, PPD model)	An 8-week HFD is used to establish a prenatal obesity model in female C57BL/6J mice	36 mg/kg body weight, every 2 days, until weaning	Barnes maze test, Y maze test, NOR, Open Field Test, Marble Burial test, Tail Suspension test, 5-HT, NE, 5-HT1AR gene expression	Restore intestinal barrier function and inhibit inflammation to prevent prenatal obesity-induced cognitive and mood disorders	Ye et al. (2024)
Epigallocatechin-3-gallate	Tea	Animals (Female mice, PPD model)	a mouse model of PPD established by exposing pregnant mice to gestational stress	50 mg/kg	OFT, FST, TST, Sema3A (ELISA), C-Fos expression (Immunofluorescence)	Downregulate Sema3A and increase GSK3β phosphorylation in the hippocampus	Xu et al. (2023)
Resveratrol	Peanuts, red grape skins and red wine	Animals (Female C57BL/6 mice, PPD model)	a mice model with bilateral oophorectomy combined with hormone-simulated pregnancy	20 mg/kg/d	OFT, FST, TST, SIRT1, ATG5, Beclin1, LC3II/I, p62 (Western blot), SIRT1 and LC3B (Immunofluorescence), mTOR, p-mTOR, AKT, p-AKT (Western blot)	Stimulate SIRT1, induce autophagy, and inhibit AKT/mTOR signaling	Ye et al. (2023b)
Sugemule-7	Nutmeg (Myristica fragrans Houtt.), Amomum (Amomum compactum Sol. ex Maton), Clove (Syzygium aromaticum (L.) Merr. and L.M.Perry), Asparagus (Asparagus cochinchinensis (Lour.) Merr.), Gymnadenia (Gymnadenia conopsea (L.) R.Br.), Agarwood (Aquilaria malaccensis Lam.), Siberian solomonseal rhizome (Polygonatum sibiricum Redouté)	Animals (Female BALB/c mice, PPD model)	HW(Hormone Withdrawal) models	Low dose: 0.325 mg/g/day; Medium dose: 0.65 mg/g/day; High dose: 1.3 mg/g/day, continuous administration during behavioral tests	SPT, OFT, FST, NSF, EPM, TST, MDA, GSH-Px, CAT, NO, SOD, T-AOC	Enhance synaptic plasticity, reduce oxidative stress, suppress inflammation, and promote neuron survival and regeneration	Fu et al. (2025b)
Shen-Qi-Jie-Yu-Fang (SJF)	Astragalus membranaceus (Fisch.) Bunge, Codonopsis pilosula (Franch.) Nannf., Ziziphus jujuba var. spinosa (Bunge) Hu ex H.F. Chow, Cornus officinalis Sieb. et Zucc., Curcuma zedoaria (Christm.) Roscoe, Citrus reticulata Blanco, Citrus medica L., Angelica sinensis (Oliv.) Diels	Animals (Female SD rats, PPD model)	Sprague Dawley rats were used to create an animal model of PPD by inducing hormone-simulated pregnancy followed by hormone withdrawal.	1.25 g/mL, body weight(g) × 0.25 mg/100 g	OFT, FST, Sucrose preference test, E, P, Cor, ACTH, CRH, 5-HT, NE, DA, IL-6, IL-10, IL-17A, Foxp3 (qPCR), CD4^+^CD25+Foxp3+ Treg cells (Flow cytometry)	Regulate serum IL-1β and IL-6 levels, hippocampal IL-1RI and gp130 expression, and peripheral CD4^+^CD25^+^ Treg cells to attenuate proinflammatory cytokine/Treg dysfunction in different stages of PPD.	Shi et al. (2023)
		Animals (Female SD rats, PPD model)	The rats were subcutaneous injected estradiol benzoate and progesterone to build PPD rat model	1.25 g/mL, 1 mL/100 g by gavage, for 3 weeks	OFT, Sucrose preference test, FST, NF-κB p65, TNF-α, IL-1β, IL-6 (ELISA)	Inhibit AA metabolism, downregulate NF-κB signaling, suppress inflammation, and increase 5-HT levels	Jingya et al. (2024)
919 granules	Actinidia chinensis, Salvia miltiorrhiza, Atractylodes macrocephala, Epimedium brevicornu, Poria cocos, Bupleurum chinense (root), Schisandra chinensis, Alpinia katsumadai, Citrus reticulata (pericarp), and Pseudostellaria heterophylla	Animals (Female BALB/c mice, PPD model)	each female in PPD group and 919 TJ group was separated from the cubs and subjected to restraint stress treatment for 3 h from 13:00 to 16:00 every day. During this time, each mouse was fixed with a plastic holder	20 mL/kg by gavage, once a day for 3 weeks	TST, FST, Body weight, Offspring survival rate, Erucamide, L-fucitol, Trimethylamine (hippocampal metabolites), Gut microbiota (16S rRNA sequencing)	Increase intestinal flora abundance (e.g., *Bacteroides*, *Lactobacillus*), decrease hippocampal erucamide, and increase hippocampal 5-AVAB.	Zheng et al. (2023)
		Animals (Female ICR mice, PPD model)	each mouse in the PPD and 919 TJ groups was separated from her pups and placed into a separate cage from 13:00 to 16:00 every day. Each mouse was immobilized during this period with a plastic retainer	20 mL/kg by gavage, once a day from PND 2 to PND 23	TST, FST, Body weight, Gut microbiota (Whole-genome shotgun sequencing), Fecal metabolites (UPLC-MS/MS)	Alter select metabolites in the hippocampus and feces	Tian et al. (2021)
		Animals (Female mice, PPD model); Humans (18 healthy postpartum women and 18 PPD patients)	were subjected to immobilization stress 21 days using immobilization device.	2.67 g/kg	TST, FST, IL-1β (ELISA in human plasma and mouse serum)	Modulate peripheral blood IL-1β to alleviate PPD. (IL-1β may be a potential therapeutic target of 919 granules.)	Wang et al. (2024)

## 6 Discussion

PPD is a common emotional disorder that poses significant risks to both the mother’s and child’s physical and mental wellbeing. Despite its prevalence, the pathophysiology of PPD remains unclear, and there are currently no fully effective intervention strategies to prevent or completely reverse the condition. These challenges represent a persistent issue in contemporary clinical practice. Preclinical evidence summarized in this review suggests the therapeutic potential of certain TCM formulations and metabolites for PPD management, primarily mediated through multiple pathways including neuroinflammation and the MGB axis. Such a multi-target approach is consistent with the principles of network pharmacology. However, translating these promising findings into clinical practice faces substantial challenges. Therefore, a key area of current research should focus on leveraging the unique advantages of TCM while compensating for the single-target limitations of conventional pharmacotherapy. Conducting in-depth research into multi-target therapies for PPD, with a goal of achieving effects similar to modern “cocktail therapy”, should be a priority. This topic is not only of scientific importance but also a growing area of social concern.

The current preclinical evidence base for many botanical drugs and their characterized metabolites in PPD has critical limitations that hinder clinical translation. This body of evidence largely depends on animal and *in vitro* models, lacks robust clinical validation, and carries insufficient safety data, particularly for lactating women. Firstly, from the perspective of research methodology, most preclinical studies exhibit deficiencies in plant material identification, extract standardization, and experimental reporting. According to the ConPhyMP guidelines (Heinrich et al., 2022), research reproducibility depends on the standardized documentation of plant species, extraction processes, and chemical composition. However, a considerable proportion of studies in this field do not adhere to these fundamental standards. This lack of adherence severely undermines the credibility and comparability of the reported findings. Additionally, the physicochemical properties of certain metabolites from these botanical drugs, such as low bioavailability, poor chemical stability, and limited BBB penetration, significantly hinder their ability to reach the CNS. Furthermore, the heterogeneity in the cultivation conditions of raw botanical materials, non-standardized processing methods, and substantial batch-to-batch variations in metabolite concentrations pose challenges for maintaining quality control. These inconsistencies not only restrict the clinical applicability of botanical drug-based interventions but also complicate efforts to understand the mechanisms underlying their putative effects.

More importantly, any bioactive substance can have both pharmacological effects and off-target effects on normal tissues (Guo et al., 2023). The metabolites derived from botanical drugs used in TCM are no exception and carry potential toxicological risks and side effects (Bernstein et al., 2021). However, comprehensive assessments of the side effects and long-term risks associated with these metabolites are still lacking in the literature, especially concerning their impact on healthy tissues when multiple constituents are combined. However, the existing literature severely lacks systematic safety assessments adhering to the ConPhyMP framework. This guideline emphasizes that toxicological assessments of plant extracts and their metabolites should be regarded as equally important as pharmacodynamic studies, particularly during the lactation period—a physiologically sensitive stage. At present, research on the off-target effects, chronic toxicity, and potential risks arising from the synergistic interactions among multiple constituents of TCM remains almost nonexistent, which constitutes one of the major barriers to its clinical translation. One of the major obstacles hindering the clinical translation of active metabolites from TCM is the absence of a systematic safety evaluation framework.

Future studies should focus on the following areas to overcome existing bottlenecks:

1. Rigorous evaluation of efficacy and safety through large-scale, multi-center, randomized double-blind placebo-controlled trials to assess the effects of characterized TCM metabolites and formulations on PPD patients; (2) Integrate single-cell sequencing, spatial multi-omics techniques, and systems biology methods to deepen multidimensional mechanism analysis, revealing the core system interaction network and multi-target mechanisms of TCM interventions; (3) Develop personalized treatment plans based on biomarkers and explore the optimization and integration of precision interventions alongside nonpharmacological interventions; (4) Development of novel intelligent targeted delivery technologies (e.g., cell-penetrating peptides or multifunctional nanocarriers with active targeting ligands) to overcome the poor CNS delivery of active TCM metabolites. (5) Establishment of a standardized quality control system covering the entire supply chain of botanical drugs. In future basic research, strict adherence to international consensus guidelines such as ConPhyMP is essential. This requires standardization at every stage, from material traceability and chemical characterization of extracts to the reporting of pharmacological and toxicological data. Such rigorous practices are fundamental for generating high-quality, reproducible evidence and laying a solid foundation for clinical translation. By integrating these strategies, it is expected that we will gain a clearer understanding of the scientific basis of TCM in preventing and treating PPD, thereby providing a theoretical foundation for the development of safe and effective new therapies, facilitating their industrial transformation and clinical application, and ultimately improving maternal and infant health outcomes.
